# Palmitoylethanolamide/Baicalin Supplementation and Changes in Pain and Sudomotor Function in Type 2 Diabetes: A Retrospective Matched Real-World Cohort Study

**DOI:** 10.3390/nu18121894

**Published:** 2026-06-11

**Authors:** Salvatore Scibetta, Luigi Calvo, Laura Pinzolo, Giacomo Corrao, Salvatore Corrao

**Affiliations:** 1 Internal Medicine Unit with Rheumatology, Dermatology, Diabetology and Tertiary Diabetic Foot Health-Care, Department of Clinical Medicine, National Relevance and High Specialization Hospital Trust ARNAS Civico, Di Cristina, Benfratelli, 90127 Palermo, Italy; salvatore.scibetta@live.it (S.S.); luigi.calvo@yahoo.it (L.C.); laurapinzolo@gmail.com (L.P.); 2 Institute for Biomedical Research and Innovation (IRIB), National Research Council (CNR), Via Ugo La Malfa 153, 90146 Palermo, Italy; 3 Department of Health Promotion Sciences, Maternal and Infant Care, Internal Medicine and Medical Specialties [PROMISE], University of Palermo, 90133 Palermo, Italy; 4 Department of Internal Medicine, Azienda Ospedaliera Universitaria “Policlinico G. Martino”, 90124 Messina, Italy; gcmcorrao@gmail.com

**Keywords:** diabetic peripheral neuropathy, Palmitoylethanolamide, Baicalin, Sudoscan, electrochemical skin conductance, VAS, nutraceutical, type 2 diabetes, pain, sudomotor function

## Abstract

**Background:** Diabetic peripheral neuropathy (DPN) is a progressive complication of type 2 diabetes mellitus (T2DM) for which no approved disease-modifying therapy exists. Palmitoylethanolamide/Baicalin (PEA/Bai; Neuridase^®^) is a nutraceutical formulation with anti-neuroinflammatory and antioxidant properties; however, real-world evidence on its associations with objective neuropathy biomarkers remains limited; nutraceutical approaches to DPN remain exploratory and adjunctive in the absence of randomised controlled trial evidence of disease modification. **Methods:** We conducted a single-centre, retrospective, 1:1 matched-cohort study at an Internal Medicine outpatient clinic. Forty-eight T2DM patients with clinically diagnosed DPN who received PEA/Bai supplementation (Neuridase^®^ group) were matched to 48 untreated controls drawn from a large institutional database, using age, sex, BMI, and diabetes duration as matching variables. Acknowledged a priori limitations include baseline imbalance in neuropathy severity (VAS and ESC) and SGLT2 inhibitor use, reflecting real-world prescribing patterns (confounding by indication) and constituting potential sources of residual confounding that preclude causal inference. The primary outcome was change in VAS neuropathic pain score from baseline (T0) to 6-month follow-up (T6). Secondary outcomes were changes in electrochemical skin conductance (ESC, µS) in hands, feet, and four-limb sum measured by Sudoscan. **Results:** At baseline, the Neuridase^®^ group exhibited significantly greater neuropathic burden: higher VAS scores (median 5.5 [IQR 3.8–7.2] vs. 2.0 [0.0–5.0]; *p* < 0.001) and lower ESC in both hands (53.0 vs. 72.2 µS; *p* < 0.001) and feet (74.5 vs. 81.0 µS; *p* < 0.001), reflecting real-world prescribing patterns. Over 6 months, VAS decreased significantly in the Neuridase^®^ group (5.5→3.0; *p* < 0.0001; median Δ = −2.5 points, exceeding the clinically important difference), with no change in controls (2.0→2.0; *p* = 0.85). Differential Sudoscan trajectories were observed: the Neuridase^®^ group showed significant improvement in hand ESC (53.0→60.0 µS; *p* = 0.035) and preservation of foot ESC (*p* = 0.888), while controls exhibited significant deterioration across all three sudomotor indices (hand *p* = 0.038; foot *p* = 0.008; four-limb sum *p* = 0.004). In a complementary categorical pain trajectory analysis, VAS worsening occurred in 31.3% of controls compared with 0% of Neuridase^®^-treated patients (*p* = 0.00022). Among patients with pathological hand ESC at baseline (<60 µS), 27.8% of Neuridase^®^ patients (n = 36) transitioned to non-pathological values at T6 versus 0% of controls (n = 32; *p* = 0.001). **Conclusions:** In a real-world matched cohort, PEA/Baicalin supplementation was associated with clinically meaningful pain reduction and with differential longitudinal sudomotor trajectories compared to matched untreated controls. These exploratory, hypothesis-generating findings from a retrospective non-randomised design are consistent with possible modulatory effects of PEA/Baicalin on objective sudomotor autonomic biomarkers in DPN. Confounding by indication, baseline severity imbalance, and residual confounders including SGLT2 inhibitor use preclude causal interpretation. These observations provide a rationale for adequately powered, prospective, randomised placebo-controlled trials with extended follow-up and structural neuropathy endpoints.

## 1. Introduction

Diabetic peripheral neuropathy (DPN) is the most prevalent long-term neurological complication of type 2 diabetes mellitus (T2DM), affecting 30–50% of individuals in unselected cohorts and exceeding 70% in patients with prolonged disease duration or suboptimal glycaemic control [1,2]. Its clinical spectrum spans asymptomatic nerve conduction slowing, chronic painful neuropathy, and progressive autonomic dysfunction, culminating in foot ulceration, lower-limb amputation, and increased mortality [3]. Beyond structural nerve damage, DPN imposes a substantial quality-of-life burden through neuropathic pain, gait instability, and autonomic symptoms, placing it among the most disabling and costly microvascular complications of diabetes [4].

The pathophysiology of DPN is multifactorial, involving chronic hyperglycaemia-driven oxidative stress, activation of the polyol and hexosamine pathways, advanced glycation end-product (AGE) accumulation, protein kinase C dysregulation, and mitochondrial dysfunction [5,6]. These converging mechanisms produce endoneural ischaemia, Schwann cell injury, axonal degeneration, and demyelination, causing characteristic small- and large-fibre damage [4,6].

Approved pharmacological agents for painful DPN—tricyclic antidepressants, serotonin–norepinephrine reuptake inhibitors (duloxetine), and anticonvulsants (pregabalin, gabapentin)—provide only symptomatic pain relief without modifying the underlying neuropathic process [7]. This persistent unmet need has stimulated interest in nutraceutical compounds targeting neuroinflammation, oxidative stress, and neural metabolic dysfunction [8,9].

Palmitoylethanolamide (PEA) is an endogenous *N*-acylethanolamine and peroxisome proliferator-activated receptor-α (PPAR-α) agonist with well-characterised anti-inflammatory and analgesic properties, acting through down-regulation of mast cell activation, reduction in glial neuroinflammation, and modulation of endocannabinoid tone [10,11]. Baicalin, a flavonoid derived from *Scutellaria baicalensis*, exerts complementary antioxidant effects and has been associated with reduction in oxidative stress and suppression of inflammatory signalling pathways including MAPK and NF-κB cascades. Clinical evidence from combination therapy including Baicalin in diabetic peripheral neuropathy has shown improvement in nerve conduction velocity together with attenuation of oxidative and inflammatory injury markers [12], while experimental studies support antioxidant and anti-inflammatory effects of Baicalin in diabetes-related complications [13]. The combination of PEA and Baicalin (commercialised as Neuridase^®^) provides mechanistically synergistic targeting of neuroinflammation and oxidative stress, the two principal drivers of small-fibre neuropathy in diabetes [14].

Non-invasive assessment of sudomotor function by electrochemical skin conductance (ESC) measured with Sudoscan (Impeto Medical, Paris, France) has emerged as a validated, sensitive biomarker of small-fibre autonomic neuropathy [15,16]. ESC values reflect sweat gland function mediated by postganglionic cholinergic *C*-fibres and correlate with nerve conduction variables, intraepidermal nerve fibre density, and clinical severity [17]. Below established reference thresholds (hands < 60 µS; feet < 70 µS), ESC reliably identifies DPN and provides an objective measure complementary to pain self-assessment [15].

Despite the biological plausibility of PEA/Baicalin supplementation in DPN, real-world evidence of its longitudinal associations with objective neuropathy biomarkers—beyond self-reported pain—remains limited. The present study aimed to evaluate, in a 1:1 matched retrospective cohort, the 6-month effect of PEA/Baicalin (Neuridase^®^) supplementation and its associations with neuropathic pain (VAS) and sudomotor function (Sudoscan ESC) in T2DM patients with clinically diagnosed DPN, using a large institutional database to construct a contemporaneous control cohort matched on key demographic and metabolic variables, within the acknowledged constraints of a non-randomised observational design aimed at generating hypotheses rather than confirming therapeutic efficacy.

## 2. Research Design and Methods

### 2.1. Study Design and Setting

This single-centre, retrospective, 1:1 matched-cohort study was conducted at the Internal Medicine and Metabolic Diseases outpatient clinic, ARNAS Civico di Palermo, University of Palermo, Italy. Data were extracted from a prospectively maintained institutional clinical database of T2DM patients visiting the clinic between January 2020 and December 2023. The study was conducted in accordance with the Declaration of Helsinki and the ethical guidelines for observational research; ethics committee approval was obtained under institutional protocol (n. 132 CIVICO 2025). The requirement for individual informed consent was waived given the retrospective nature of data collection and de-identification of the dataset.

### 2.2. Study Population and Eligibility

Patients were eligible if they met all of the following criteria: (i) confirmed T2DM diagnosis per American Diabetes Association (ADA) criteria; (ii) age ≥ 18 years; (iii) clinical diagnosis of DPN, established on the basis of characteristic symptoms (burning, allodynia, paraesthesia, numbness), neurological examination, and objective Sudoscan measurement; and (iv) complete baseline (T0) and 6-month follow-up (T6) data, including VAS score and Sudoscan ESC. Exclusion criteria were: type 1 diabetes; secondary causes of peripheral neuropathy (alcohol-related, hypothyroid, chemotherapy-induced, or hereditary); end-stage renal disease (eGFR < 15 mL/min/1.73 m^2^); active neoplastic disease; concurrent use of other nutraceutical supplements with established neuropathic effects; inability to perform Sudoscan; and incomplete records at either visit.

### 2.3. Cohort Construction and Matching Procedure

From the eligible population, 48 patients who received PEA/Baicalin (Neuridase^®^) supplementation at the T0 visit constituted the treated cohort. A control cohort of 48 patients who received no nutraceutical supplementation was constructed by 1:1 nearest-neighbour matching without replacement, drawn from the full institutional database of eligible T2DM patients. Matching variables were: age (years), sex, BMI (kg/m^2^), and diabetes duration (months), selected as the principal determinants of DPN severity and natural history [1,3]. Balance after matching was assessed by standardised mean differences (SMDs); SMD < 0.10 was pre-specified as acceptable balance. No significant differences were observed for any matching variable (all *p* > 0.10), confirming adequate cohort balance (Table 1). Standardised mean differences (SMDs) were ≤0.10 for all four matching variables; the SGLT2 inhibitor imbalance (SMD = 0.25) persisted as the principal pre-specified residual confounder and is addressed in Section 4.

### 2.4. Intervention

Patients in the Neuridase^®^ group received one tablet twice a day of oral PEA/Baicalin supplementation continuously for 6 months (T0 to T6). Each dose of Neuridase^®^ contains 600 mg of Palmitoylethanolamide and 250 mg of Baicalin. Concomitant pharmacological management of diabetes, dyslipidaemia, and hypertension was continued according to clinical judgement without protocol-mandated modifications. Adherence was evaluated through review of scheduled follow-up clinical records, documentation of ongoing prescription use at follow-up visits, and absence of treatment discontinuation during the 6-month observation period. We acknowledge that chart-review adherence assessment is inherently less robust than formal pill-count or pharmacokinetic verification, a limitation explicitly noted in Section 4. Concomitant use of first-line neuropathic pain medications (pregabalin, duloxetine, tricyclic antidepressants) was recorded; dosages remained stable throughout the observation period in patients receiving such treatment, and changes in concomitant analgesic therapy during follow-up cannot be completely excluded as an additional source of residual confounding.

### 2.5. Outcome Measures

**Primary outcome:** Change in VAS neuropathic pain score from T0 to T6. The VAS is a validated 0–10 numerical rating scale (0: no pain; 10: worst imaginable pain), self-administered by the patient at each scheduled visit under standardised conditions.

**Secondary outcomes:** Change in sudomotor function from T0 to T6 assessed by Sudoscan (Impeto Medical, Paris, France), an operator-independent device that measures ESC in microsiemens (µS) at the palms and plantar soles through low-voltage direct current applied to large stainless-steel electrodes. Four individual ESC values were obtained per patient (left hand, right hand, left foot, right foot); mean hand ESC, mean foot ESC, and total four-limb ESC sum were computed as pre-specified composite sudomotor indices. Measurements were performed by a trained technician under standardised conditions (room temperature 20–24 °C, resting period ≥ 10 min, following the published Impeto Medical protocol).

### 2.6. Covariate Collection

The following clinical and laboratory data were extracted at T0: age, sex, height, body weight, BMI, waist circumference, diabetes duration, fasting plasma glucose, HbA1c, serum creatinine (with eGFR by CKD-EPI equation), LDL cholesterol, presence of hepatic steatosis (assessed by ultrasound with standardised grading: mild/moderate/severe), arterial hypertension, dyslipidaemia, ischaemic heart disease, cerebrovascular disease, carotid atherosclerosis (by duplex ultrasound), hypothyroidism, and carpal tunnel syndrome. Complete antidiabetic and cardioprotective medication lists were recorded, including SGLT2 inhibitors, GLP-1 receptor agonists, metformin, insulin, and statins.

### 2.7. Statistical Analysis

Continuous variables are reported as median and interquartile range (IQR), given non-normal distributions confirmed by Shapiro–Wilk test for all key variables. Categorical variables are expressed as counts and proportions. Between-group comparisons at baseline used the Mann–Whitney U test for continuous data and the chi-squared test or Fisher’s exact test for categorical data. Intragroup changes from T0 to T6 were analysed by the Wilcoxon signed-rank test on paired observations, with all paired values available in both groups. All tests were two-tailed; statistical significance was set at *p* < 0.05. Analyses were performed using Python (version 3.11; SciPy library, version 1.11). No imputation was applied; all enrolled patients had complete paired records at both time points. Exploratory adjusted analyses—including ANCOVA with baseline VAS as covariate and sensitivity analyses stratified by SGLT2 inhibitor use—were explored but deemed potentially unstable and subject to overfitting given the limited sample size (n = 48 per group), substantial baseline imbalance, complete-case constraints, and subgroup fragmentation; these are not formally presented. To improve clinical interpretability while minimising overfitting risk, two complementary categorical analyses were pre-specified: (1) a longitudinal pain trajectory analysis classifying patients as experiencing VAS worsening or non-worsening at T6; and (2) a threshold–transition analysis for hand ESC using the validated clinical cutpoint of 60 µS, restricted to patients with pathological ESC at baseline (<60 µS). Between-group differences in categorical outcomes were assessed by Fisher’s exact test.

## 3. Results

### 3.1. Baseline Characteristics

After 1:1 matching, 96 patients were included: 48 in the Neuridase^®^ group and 48 in the control group. Matching was successful across all four matching variables: age (median 71.0 [IQR 60.0–76.0] vs. 68.0 [60.0–75.0] years; *p* = 0.540), sex (29.2% vs. 41.7% female; *p* = 0.286), BMI (28.3 [26.2–32.6] vs. 29.3 [26.9–33.0] kg/m^2^; *p* = 0.439), and diabetes duration (144 [54–252] vs. 108 [36–198] months; *p* = 0.120).

The two cohorts were also balanced for the majority of clinical and biochemical variables, including body weight, waist circumference, fasting glucose, HbA1c, creatinine, LDL cholesterol, prevalence of arterial hypertension, dyslipidaemia, hepatic steatosis, ischaemic heart disease, and use of metformin, GLP-1 receptor agonists, statins, and insulin (all *p* > 0.05; Table 1). One pre-specified covariate showed a significant imbalance: SGLT2 inhibitor use was more frequent in the Neuridase^®^ group (50.0% vs. 25.0%; *p* = 0.020), a difference that persisted despite matching and is addressed in Section 4.

The distribution of neuropathic severity at baseline was, as expected from a clinically driven prescribing pattern, substantially greater in the Neuridase^®^ group. VAS pain score was markedly higher (median 5.5 [IQR 3.8–7.2] vs. 2.0 [0.0–5.0]; *p* < 0.001), while mean hand ESC was lower (53.0 [45.0–61.5] vs. 72.2 [64.5–77.5] µS; *p* < 0.001, below the 60 µS clinical threshold for moderate dysfunction), and mean foot ESC was lower (74.5 [63.4–81.1] vs. 81.0 [76.4–84.5] µS; *p* < 0.001; Table 1). This pattern is consistent with confounding by indication—the principal threat to internal validity in the present study—whereby supplementation was preferentially offered to patients with greater symptomatic burden and objectively documented neuropathy. The resulting non-comparability of baseline neuropathic severity is explicitly acknowledged as a primary limitation, and the possibility that regression to the mean and natural pain fluctuation contributed to the observed VAS reduction in the treated group cannot be excluded.

### 3.2. Primary Outcome: VAS Neuropathic Pain Score

**Figure 1:**
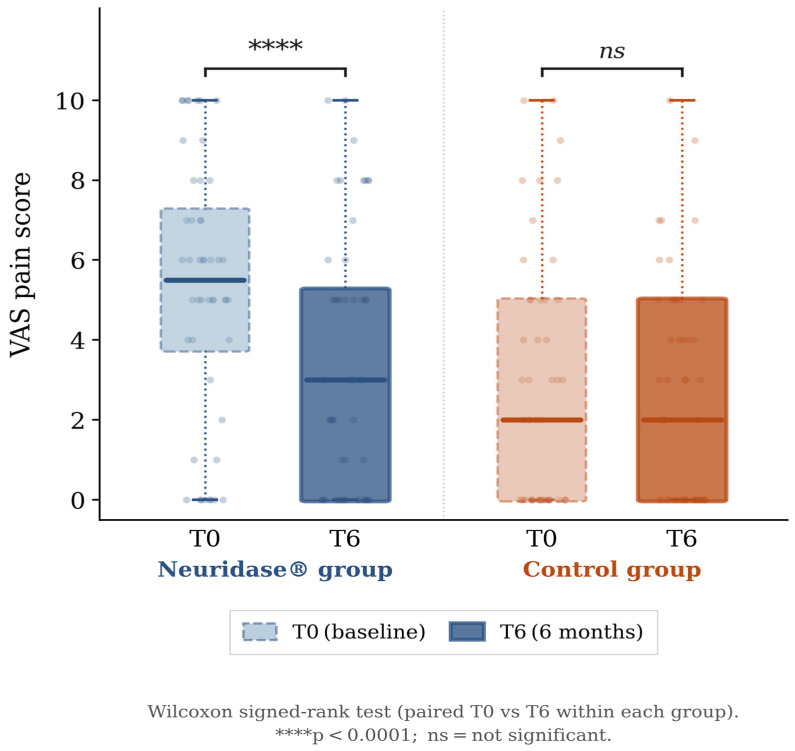
VAS Neuropathic Pain Score—Neuridase^®^ group vs. control group (n = 48 each). VAS neuropathic pain score at baseline (T0) and 6-month follow-up (T6) in the Neuridase^®^ group and control group (n = 48 per group). Box-and-whisker plots show median (horizontal line), interquartile range (box), and 1.5 × IQR (whiskers); individual patient values shown as jittered dots. Brackets indicate intragroup comparisons by Wilcoxon signed-rank test (paired T0 vs. T6). VAS, Visual Analogue Scale (0–10; 0, no pain; 10, worst imaginable pain). The Neuridase^®^ group shows a statistically significant, clinically meaningful reduction in pain score at T6 (median Δ = −2.5 points), while the control group remains unchanged.

In the Neuridase^®^ group, VAS pain score decreased significantly from baseline to 6 months (median 5.5 [IQR 3.8–7.2] at T0 vs. 3.0 [0.0–5.25] at T6; *p* < 0.0001, Wilcoxon signed-rank), representing a median absolute reduction of 2.5 points—well exceeding the minimum clinically important difference of 1.5 points established for numerical pain ratings in DPN (Figure 1). In the control group, VAS scores were low at baseline and showed no significant change over the observation period (2.0 [0.0–5.0] at T0 vs. 2.0 [0.0–5.0] at T6; *p* = 0.85; Figure 1).

The magnitude of within-group change in the Neuridase^®^ group no longer showed a statistically significant between-group difference in VAS at 6 months (*p* = 0.136); this convergence from substantially different baselines reflects the observational nature of the data and cannot be interpreted as evidence of treatment efficacy independent of regression to the mean. The possibility that regression to the mean partially contributed to this reduction—given that the treated group was selected, in part, based on higher baseline pain scores—cannot be excluded in the absence of a formally adjusted analysis. To address this concern using a clinically interpretable categorical approach, a complementary longitudinal pain trajectory analysis was conducted. VAS worsening during follow-up occurred in 31.3% of controls, whereas no patient receiving Neuridase^®^ experienced worsening pain score over the 6-month period (0%; Fisher’s exact test: *p* = 0.00022). This differential categorical trajectory—directionally consistent with the continuous VAS reduction and independent of absolute baseline level—provides complementary support for the observed longitudinal pain divergence while acknowledging the inherent interpretive constraints of the observational design.

### 3.3. Secondary Outcomes: Sudomotor Function

**Figure 2:**
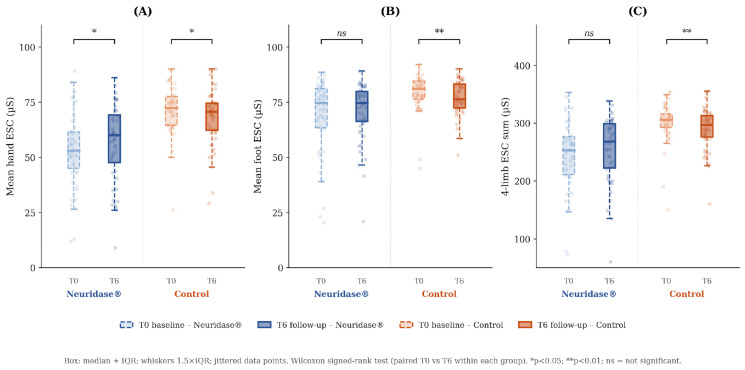
Sudoscan ESC: Panels (**A**) hands, (**B**) feet, (**C**) 4-limb sum—Neuridase^®^ group vs. control group (n = 48 each). Sudoscan electrochemical skin conductance (ESC, µS) at baseline (T0) and 6-month follow-up (T6) in the Neuridase^®^ group and control group (n = 48 per group), shown as intragroup paired comparisons. Panels: (**A**) mean hand ESC; (**B**) mean foot ESC; (**C**) total four-limb ESC sum. Box-and-whisker plots display median (horizontal line), IQR (box), 1.5 × IQR (whiskers), and jittered individual data points. Lighter shaded boxes (dashed border) represent T0; darker solid boxes represent T6. Significance brackets reflect Wilcoxon signed-rank test for each within-group paired comparison. The Neuridase^®^ group shows significant improvement in hand ESC ((**A**); *p* = 0.035) and stable foot ESC and four-limb sum, while the control group demonstrates significant deterioration across all three sudomotor indices (hand *p* = 0.038; foot *p* = 0.008; four-limb sum *p* = 0.004), consistent with progressive longitudinal sudomotor deterioration observed during follow-up. ESC, electrochemical skin conductance; IQR, interquartile range.

Mean hand ESC (Figure 2A): In the Neuridase^®^ group, mean hand ESC improved significantly from T0 to T6 (median 53.0 [45.0–61.5] vs. 60.0 [47.6–69.1] µS; *p* = 0.035), representing a median gain of 7.0 µS—a change that crossed the clinically validated hand ESC threshold of 60 µS. The control group showed a significant deterioration in hand ESC over the same period (72.2 [64.5–77.5] vs. 70.5 [62.2–74.5] µS; *p* = 0.038).

Mean foot ESC (Figure 2B): In the Neuridase^®^ group, mean foot ESC remained stable (74.5 [63.4–81.1] vs. 74.5 [66.2–79.8] µS; *p* = 0.888). The control group showed a significant decrease from T0 to T6 (81.0 [76.4–84.5] vs. 76.2 [72.4–83.1] µS; *p* = 0.008).

Four-limb ESC sum (Figure 2C): Total four-limb ESC sum showed no significant change in the Neuridase^®^ group (253.0 [210.8–276.8] vs. 268.0 [222.5–298.8] µS; *p* = 0.151), but declined significantly in the control group (305.5 [292.5–315.8] vs. 296.5 [276.0–313.0] µS; *p* = 0.004). Taken together, these data describe a notable divergence of sudomotor biomarker trajectories between groups: the Neuridase^®^ group maintained or improved ESC values across all three indices, while the control group deteriorated significantly on each measure, a pattern consistent with differential longitudinal autonomic biomarker trajectories between the two groups. Whether this divergence reflects a modulatory effect of PEA/Baicalin supplementation, differences in baseline disease trajectory, SGLT2 inhibitor co-treatment, or other unmeasured factors cannot be established from the present observational data.

### 3.4. Clinically Validated Hand ESC Threshold–Transition Analysis

To further improve the clinical interpretability of continuous ESC trajectory analyses, we additionally explored categorical transition across clinically validated sudomotor dysfunction thresholds. Because hand ESC values < 60 µS are considered indicative of pathological sudomotor impairment in diabetic peripheral neuropathy, this analysis was intentionally restricted to patients presenting pathological hand ESC values at baseline.

**Figure 3:**
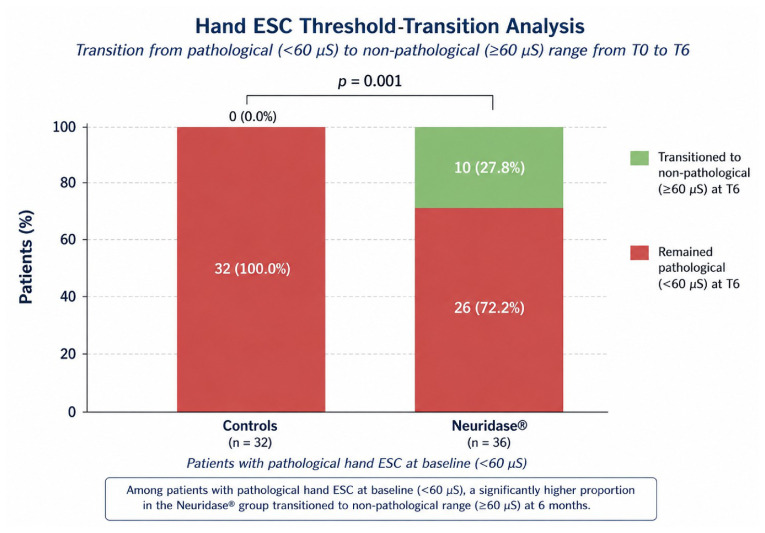
Hand ESC threshold–transition analysis among patients with pathological baseline hand ESC values (<60 µS). Stacked bar plots illustrate the proportion of patients who either remained within the pathological sudomotor range (<60 µS) or transitioned to the non-pathological range (≥60 µS) after 6 months (T6). The analysis was intentionally restricted to patients with pathological hand electrochemical skin conductance (ESC) values at baseline, corresponding to clinically validated thresholds for sudomotor dysfunction in diabetic peripheral neuropathy. In the control group, no patient transitioned to the non-pathological range during follow-up (0/32, 0%), whereas 10/36 patients (27.8%) receiving Neuridase^®^ transitioned above the clinically validated pathological threshold (*p* = 0.001). Because ESC represents an operator-independent objective autonomic biomarker, this threshold–transition analysis provides a clinically interpretable complement to continuous ESC trajectory analyses. ESC, electrochemical skin conductance.

As shown in Figure 3, none of the control patients transitioned from the pathological to the non-pathological range during the 6-month follow-up period (0/32, 0%), whereas 10/36 patients (27.8%) receiving Neuridase^®^ transitioned above the clinically validated pathological threshold (≥60 µS; *p* = 0.001). Given that ESC represents an operator-independent, objective autonomic biomarker, this analysis provides a clinically meaningful complement to subjective pain assessment and supports the presence of differential longitudinal sudomotor trajectories between groups.

Importantly, the observed threshold–transition pattern was directionally coherent with the continuous ESC analyses and with the parallel stabilisation of pain trajectories observed in the treated cohort. Although the observational nature of the study precludes definitive causal inference, the convergence between objective autonomic findings and clinical symptom evolution strengthens the biological plausibility of a potential modulatory effect on small-fibre dysfunction and supports the rationale for future prospective randomised investigations incorporating structural neuropathy endpoints.

## 4. Discussion

This retrospective, 1:1 matched-cohort study reports two interrelated exploratory observations in a real-world T2DM population with clinically diagnosed DPN. First, 6-month supplementation with Palmitoylethanolamide/Baicalin (PEA/Bai; Neuridase^®^) was associated with a statistically significant, clinically meaningful reduction in neuropathic pain (median VAS Δ = −2.5 points; *p* < 0.0001), substantially exceeding the established minimum clinically important difference of 1.5 points for numerical pain ratings in DPN. Second, differential longitudinal trajectories of sudomotor function were observed between groups: while controls demonstrated progressive and statistically significant deterioration across all three Sudoscan indices over the same 6-month window, the Neuridase^®^ group showed both significant improvement in hand ESC and preservation of foot ESC. A complementary threshold–transition analysis further demonstrated that 27.8% of Neuridase^®^ patients with pathological hand ESC at baseline transitioned to non-pathological values at T6 versus 0% of controls (*p* = 0.001). These observations are presented as hypothesis-generating signals from an observational cohort rather than as evidence of treatment efficacy or disease modification.

The pain-related signal observed in the Neuridase^®^ group is mechanistically coherent and consistent with the pharmacological profile of both active components. PEA operates principally as an endogenous PPAR-α agonist: receptor activation suppresses the transcription of NF-κB-regulated pro-inflammatory mediators including TNF-α, IL-1β, COX-2, and inducible nitric oxide synthase in peripheral immune cells, resident macrophages, and spinal microglia, thereby attenuating both peripheral sensitisation and central pain amplification [10]. Concomitantly, PEA stabilises mast cell degranulation and reduces release of histamine and nerve growth factor at the level of endoneurial tissue, dampening the neuro-immune crosstalk that perpetuates neuropathic hypersensitivity in DPN [11,14]. PEA additionally inhibits fatty acid amide hydrolase (FAAH), the principal enzyme responsible for the hydrolytic degradation of endogenous anandamide. This competitive inhibition reduces anandamide catabolism and prolongs its biological activity, thereby amplifying endocannabinoid signalling through CB1 and CB2 receptors and contributing an additional pain-modulatory dimension through modulation of nociceptive transmission and suppression of neuroinflammatory cascades—the so-called “entourage effect” of PEA. Baicalin complements these effects through a distinct but convergent pathway. Experimental studies indicate activation of Nrf2-dependent antioxidant responses together with inhibition of MAPK- and NF-κB-mediated inflammatory signalling, reducing oxidative tissue injury in diabetes-related complications [13]. In addition, clinical evidence from combination therapy including Baicalin suggests improvement in nerve conduction parameters and attenuation of inflammatory burden in patients with diabetic peripheral neuropathy [12]. This dual mechanism—PPAR-α-mediated neuro-immune modulation combined with antioxidant and anti-inflammatory signalling effects of Baicalin—addresses two of the principal molecular drivers of neuropathic pain in T2DM, providing a biologically plausible mechanistic framework for the observed longitudinal pain score divergence within an acknowledged observational context.

The pattern of sudomotor function changes over 6 months warrants careful interpretation in the context of trajectory divergence rather than simple between-group comparisons. The natural history of small-fibre autonomic neuropathy in T2DM is one of progressive decline: postganglionic cholinergic *C*-fibres mediating sweat gland function are among the earliest neural elements lost in DPN, and their progressive dropout produces the falling ESC values routinely observed in longitudinal cohorts of T2DM patients under standard care [3,6]. The control group in the present study exhibited exactly this expected pattern, with statistically significant deterioration in hand ESC (*p* = 0.038), foot ESC (*p* = 0.008), and total four-limb ESC sum (*p* = 0.004) over just 6 months, a finding that contextualises the trajectory of the treated group. The Neuridase^®^ group, starting from substantially more impaired baseline ESC values, and therefore at greater risk of measurable decline, instead showed significant improvement in hand ESC (+7.0 µS; *p* = 0.035) and stable foot ESC. The resulting divergence of trajectories—improvement or stabilisation in treated patients versus progressive deterioration in matched controls—is biologically plausible in light of the pharmacological properties described above [10,13], though the observational design does not permit causal attribution. The anatomical gradient of the response—with greater improvement at the hands than at the feet—is also biologically plausible: hand ESC in the Neuridase^®^ group was below the 60 µS clinical threshold at baseline (53.0 µS), representing a zone of objectively documented sudomotor impairment in which transition across a validated clinical threshold was operationally demonstrable within a 6-month window, while the feet—more severely involved in the most compromised patients—may require a longer time-horizon to demonstrate measurable functional changes. What the data demonstrate is differential objective autonomic biomarker behaviour across the observation period; structural nerve fibre regeneration cannot be inferred from functional ESC changes alone.

An important covariate imbalance merits transparent and nuanced treatment. SGLT2 inhibitor use was significantly more prevalent in the Neuridase^®^ group (50.0% vs. 25.0%; *p* = 0.020), persisting despite matching on the four pre-specified variables. SGLT2 inhibitors have been associated with improvements in neuropathic pain and sudomotor function in observational analyses [18], and this imbalance represents a substantive potential confounder not formally adjusted for in the primary analyses. We explored adjusted sensitivity analyses incorporating SGLT2 inhibitor exposure; however, because of the limited sample size and subgroup fragmentation after stratification, we considered these analyses insufficiently stable for definitive inference. It is noteworthy that, despite the higher prevalence of SGLT2 inhibitor use, the Neuridase^®^ group exhibited significantly worse ESC values at baseline, suggesting that SGLT2 inhibitor exposure alone was insufficient to account for the greater neuropathic burden observed at study entry. This observation does not exclude potential contribution from PEA/Baicalin supplementation to the subsequent longitudinal divergence in autonomic biomarker trajectories, although residual confounding related to concomitant therapies, baseline clinical differences, and other unmeasured factors cannot be completely excluded within the current retrospective observational design. Future prospective studies should incorporate stratified analyses according to SGLT2 inhibitor exposure and additional neuropathy-related co-therapies. Prospective studies must incorporate SGLT2 inhibitor status as a pre-specified stratification variable and collect complete concomitant treatment data.

Several features of the study design support the validity of the findings. The use of a large institutional database for control selection enabled contemporaneous real-world comparisons, minimising temporal confounding inherent in historical controls. Matching achieved excellent balance across majority of the clinical and biochemical variables, including glycaemic indices, lipid profile, renal function, and comorbidity burden. All outcomes were assessed using validated, standardised instruments under controlled conditions by trained personnel; in particular, the Sudoscan-derived ESC provides an operator-independent, highly reproducible objective biomarker of small-fibre autonomic function, free from the expectation bias and placebo susceptibility that affect self-reported VAS scores. The convergence of the objective threshold–transition analysis—showing differential threshold–transition rates across a clinically validated cutpoint—with the continuous ESC trajectory findings, the categorical pain trajectory divergence (31.3% vs. 0% VAS worsening; *p* = 0.00022), and the continuous pain reduction provides multi-dimensional observational consistency, strengthening biological plausibility without establishing causality. Crucially, 100% paired outcome data availability across both time points eliminates attrition bias.

The baseline disparity in neuropathic severity reflects real-world prescribing behaviour (confounding by indication) and constitutes the principal threat to internal validity. The convergence of VAS scores between groups at T6—from substantially different baseline levels—is noted but cannot be interpreted as evidence of treatment efficacy independent of regression to the mean and natural symptom fluctuation.

The limitations of this study are those inherent to the retrospective matched-cohort design and are acknowledged with proportionate candour. The observational structure precludes causal inference; the association between PEA/Baicalin supplementation and the observed outcomes may be affected by residual confounding from unmeasured variables, and the prescribing physician’s clinical judgement in initiating supplementation constitutes confounding by indication, which matching can attenuate but not eliminate. The SGLT2i imbalance, as discussed, is the principal identified source of residual confounding; regression to the mean in VAS scores represents an additional uncontrolled bias that cannot be formally excluded without an ANCOVA adjusted for baseline VAS. The subjective nature of VAS as primary endpoint in an unblinded non-randomised design introduces susceptibility to expectation bias and natural pain fluctuation, further constraining interpretation. The single-centre origin and cohort size (n = 48 per group), determined by real-world database availability rather than formal prospective power calculation, limit generalisability and the precision of effect-size estimation. Adherence assessment was based on clinical record review rather than formal pill counts or pharmacokinetic verification, introducing uncertainty regarding actual supplementation exposure. Changes in concomitant analgesic therapy during follow-up cannot be completely excluded as an additional source of residual confounding. No standardised quality-of-life instrument (e.g., EuroQol-5D, sleep interference, daily activity scores) was available in the database, preventing assessment of health-related quality of life. Importantly, Sudoscan ESC reflects functional sudomotor activity mediated by postganglionic cholinergic *C*-fibres rather than direct structural nerve regeneration; the 6-month follow-up is likely sufficient to detect early functional autonomic changes but remains insufficient to evaluate structural small-fibre regeneration. Future prospective randomised studies should therefore incorporate: longer follow-up (12–24 months); intraepidermal nerve fibre density (IENFD); corneal confocal microscopy (CCM), a validated non-invasive marker of early small-fibre damage in diabetic peripheral neuropathy [19]; nerve conduction studies; and validated patient-reported outcome measures. A completed STROBE checklist is provided as Supplementary Material.

These findings carry meaningful clinical implications for a disorder with no approved disease-modifying therapy. PEA/Baicalin offers a mechanistically coherent dual rationale—pain modulation through neuro-immune signalling and possible antioxidant protection of small-fibre autonomic pathways—as a safe and accessible complementary nutraceutical approach. The real-world longitudinal autonomic divergence observed across two independent outcome dimensions—continuous ESC trajectories and clinically validated threshold transitions—together with the differential categorical pain trajectory, provides hypothesis-generating evidence of sufficient biological plausibility to justify a formally powered prospective randomised placebo-controlled trial. Such a trial should incorporate longer follow-up (≥12 months), pre-specified stratification by SGLT2i status, and a composite neuropathy endpoint encompassing validated patient-reported pain outcomes, nerve conduction studies, intraepidermal nerve fibre density, and serial Sudoscan measurements.

## 5. Conclusions

In this real-world 1:1 matched-cohort study of T2DM patients with diabetic peripheral neuropathy, 6-month supplementation with PEA/Baicalin (Neuridase^®^) was associated with a statistically significant within-group reduction in neuropathic pain (median VAS Δ = −2.5 points; *p* < 0.0001) and with differential longitudinal sudomotor biomarker trajectories across all three Sudoscan indices compared to matched untreated controls. A clinically validated threshold–transition analysis demonstrated that 27.8% of Neuridase^®^ patients with pathological hand ESC at baseline transitioned to non-pathological values at T6 versus 0% of controls (*p* = 0.001), and a categorical pain trajectory analysis showed VAS worsening in 31.3% of controls versus 0% of treated patients (*p* = 0.00022). These exploratory, hypothesis-generating findings are consistent with possible modulatory effects of PEA/Baicalin on neuropathic pain and objective sudomotor autonomic biomarker trajectories in DPN. They do not establish causality, disease modification, or neuroprotection. These observations support biological plausibility and provide a rationale for adequately powered prospective randomised placebo-controlled trials with extended follow-up (≥12 months), pre-specified stratification by SGLT2i status, and a composite endpoint incorporating validated patient-reported outcomes, nerve conduction studies, intraepidermal nerve fibre density, corneal confocal microscopy, and serial sudomotor measurements.

## Figures and Tables

**Table 1:** Baseline clinical characteristics by treatment group.

Variable	Neuridase^®^ Group (n = 48)	Control Group (n = 48)	*p* Value
**Demographics and anthropometrics**			
Female sex, n (%)	14 (29.2%)	20 (41.7%)	0.286
Age (years)	71.0 (60.0–76.0)	68.0 (60.0–75.0)	0.540
Weight (kg)	80.0 (74.0–92.0)	82.5 (72.8–98.8)	0.655
BMI (kg/m^2^)	28.3 (26.2–32.6)	29.3 (26.9–33.0)	0.439
Waist circumference (cm)	108.0 (99.8–116.0)	108.5 (99.0–120.2)	0.939
**Metabolic variables**			
Fasting glucose (mg/dL)	112.0 (95.0–143.5)	116.0 (99.5–136.5)	0.865
HbA1c (%)	6.6 (6.2–7.5)	6.7 (6.1–7.5)	0.853
Creatinine (mg/dL)	0.9 (0.8–1.3)	0.9 (0.8–1.3)	0.503
LDL cholesterol (mg/dL)	86.5 ± 41.3	86.5 ± 35.5	0.997
Diabetes duration (months)	144 (54–252)	108 (36–198)	0.120
**Comorbidities**			
Type 2 diabetes, n (%)	47 (97.9%)	47 (97.9%)	—
Arterial hypertension, n (%)	33 (68.8%)	35 (72.9%)	0.822
Dyslipidaemia, n (%)	41 (85.4%)	41 (85.4%)	—
Hepatic steatosis, n (%)	33 (68.8%)	36 (75.0%)	0.650
Ischaemic heart disease, n (%)	19 (39.6%)	19 (39.6%)	—
**Pharmacological treatment**			
Statin, n (%)	36 (75.0%)	39 (81.2%)	0.621
Metformin, n (%)	33 (68.8%)	36 (75.0%)	0.650
GLP-1 receptor agonist, n (%)	23 (47.9%)	24 (50.0%)	—
SGLT2 inhibitor, n (%) †	24 (50.0%)	12 (25.0%)	0.020
Insulin, n (%)	11 (22.9%)	9 (18.8%)	0.802
**Neuropathy severity at baseline (T0)**			
VAS pain score	5.5 (3.8–7.2)	2.0 (0.0–5.0)	<0.001
Hands ESC (µS)	53.0 (45.0–61.5)	72.2 (64.5–77.5)	<0.001
Feet ESC (µS)	74.5 (63.4–81.1)	81.0 (76.4–84.5)	<0.001

Continuous data are expressed as median (IQR) unless otherwise indicated; LDL cholesterol expressed as mean ± SD. Categorical data as n (%). Between-group comparisons by Mann–Whitney U test (continuous) or χ^2^/Fisher’s exact test (categorical). ESC, electrochemical skin conductance; VAS, Visual Analogue Scale. † Pre-specified imbalance persisting after matching; see Section 4.

## Data Availability

The data supporting the findings of this study are available from the corresponding author upon reasonable request.

## Supplementary Materials

The following supporting information can be downloaded at: https://www.mdpi.com/article/10.3390/nu18121894/s1, Table S1: STROBE checklist.
