# Supplementary material for: Palmitoylethanolamide/Baicalin Supplementation and Changes in Pain and Sudomotor Function in Type 2 Diabetes: A Retrospective Matched Real-World Cohort Study
Source: Nutrients. 2026 Jun 11;18(12):1894. doi: 10.3390/nu18121894 (PMC13304978; doi:10.3390/nu18121894)
Supplement: Supplementary file 1 [file nutrients-18-01894-s001.zip › nutrients-4335725-supplementary.pdf]

STROBE-nut Checklist (Completed)

Item	Description	Reported on page(s)
1a-b	Study design indicated in title and abstract	Pages 1–2
nut-1	Nutritional exposure/supplement described	Pages 1–2
2	Background and rationale	Pages 2–3
3	Objectives and hypotheses	Page 3
4	Study design	Pages 3–4
5	Setting and dates	Page 3
nut-5	N/A	N/A
6a	Eligibility criteria and participant selection	Pages 3–4
6b	Matching criteria	Page 4
nut-6	Nutritional characteristics used for selection	See Methods
7	Outcomes, exposures, confounders	Pages 5–6
nut-7.1	Supplement composition reported	Page 5
nut-7.2	N/A	N/A
8	Data sources and measurements	Pages 5–6
nut-8.1	Assessment of supplement exposure	Page 5
nut-8.2 to 8.6	N/A	N/A
9	Bias	Pages 7, 12–13
10	Study size	Pages 6, 13
11	Quantitative variables	Pages 5–6
12	Statistical methods	Page 6
13	Participants	Pages 6–7
14	Descriptive data	Pages 4–7
15	Outcome data	Pages 7–10
16	Main results	Pages 7–10
17	Other analyses	Pages 8–10
18	Key results	Pages 10–14
19	Limitations	Page 13
20	Interpretation	Pages 10–14
21	Generalisability	Pages 13–14
22	Funding	Page 14
nut-22.1	Ethics approval	Page 3
nut-22.2	Supplementary material	Page 13
